# Medical and dental student knowledge about COVID-19 and influenza vaccines impact opinions about vaccine advocacy and future practice

**DOI:** 10.3389/fpubh.2024.1388894

**Published:** 2024-05-22

**Authors:** Victoria C. Lucia, Ana Karina Mascarenhas, Arati Kelekar, Nelia M. Afonso

**Affiliations:** ^1^Department of Foundational Medical Studies, Oakland University William Beaumont School of Medicine, Rochester, MI, United States; ^2^Prevention International, Fort Lauderdale, FL, United States; ^3^Department of Internal Medicine, Corewell Health William Beaumont University Hospital, Royal Oak, MI, United States

**Keywords:** students, influenza vaccines, COVID-19 vaccines, knowledge, opinions, vaccine hesitancy, vaccine advocacy

## Abstract

**Introduction:**

The World Health Organization has identified vaccine hesitancy as a global public health challenge. Healthcare providers are among the most influential and trusted figures for vaccine counseling. This article focuses on COVID-19 and influenza personal immunization behaviors, vaccine knowledge and opinions, and vaccine counseling confidence among future healthcare providers – dental and medical students.

**Methods:**

A cross-sectional anonymous online survey was conducted at four dental schools and one allopathic medical school in the United States. Items included personal vaccination status for the COVID-19 and influenza vaccines and vaccine-specific items developed based on past research to assess knowledge, opinions, and behaviors.

**Results:**

Two hundred and thirty-two medical and 221 dental students completed the survey. 68 and 55% scored average/above-average knowledge on COVID-19 and influenza vaccine items, respectively. There were significant differences between those with average/above-average and below-average knowledge scores regarding learning about, recommending, and advocating for vaccines and counseling vaccine-hesitant patients for both vaccines (*p* < 0.0001). Although higher-knowledge students had higher vaccination rates (*p* < 0.0001), many had insufficient knowledge about vaccines.

**Discussion:**

Healthcare providers play a crucial role in vaccine advocacy. The identified knowledge gaps are significant as they impact quality of patient care. And opinions about future vaccination practice such as recommending, providing, and counseling about vaccines. Equipping students with knowledge and communication skills will enable them to be strong vaccine advocates to improve overall public health.

## Introduction

Vaccines - the most important medical advancement in history, credited with saving millions of lives the world over, however vaccine hesitancy is a global challenge. Vaccine hesitancy, or the reluctance to receive recommended vaccination because of concerns and doubts about vaccines, was identified by the World Health Organization as one of the top 10 threats to global health in 2019 (1). Physicians and other healthcare providers (HCP) are still among the most influential and trusted figures when it comes to vaccine counseling.

Medical and dental students constitute a high-risk group for several vaccine-preventable diseases, especially COVID-19 and influenza. In addition, dentists are at a higher risk of occupational exposure due to the nature of the services provided in dental clinics.

Dentists and physicians also need to be strong promoters of vaccine confidence in their patients. The importance of HCP’s vaccine recommendations in patient and parent decisions about the vaccine has been well documented (2–6). However, to do this well, healthcare providers themselves need to be confident about the safety, effectiveness, and importance of vaccination (7). The COVID-19 pandemic highlighted the advantage of educating healthcare providers other than physicians to counsel and administer vaccines during a public health crisis. In 2020 the American Dental Association House of Delegates passed Resolution 91H-2020 that expanded the scope of practice to include vaccine administration and counseling (8). Information on vaccination factors associated with vaccination, vaccination knowledge, and confidence in counseling among future healthcare providers, specifically dental and medical students is limited. This article focuses on personal immunization behaviors, vaccine knowledge, attitudes, and confidence in vaccine counseling among dental and medical students in an effort to improve healthcare professional student education about vaccines for the benefit of overall public health. We surveyed students on 3 commonly used vaccines that should be recommended by both medical and dental providers: HPV, Influenza, and COVID-19. Findings regarding HPV are reported elsewhere (9). This article reports on the findings regarding 2 vaccines – the COVID-19 and Influenza vaccines. We hypothesize that medical and dental students with better vaccine knowledge will have more positive opinions about vaccines and future vaccine-specific behaviors in the patient care setting.

## Methods

An cross-sectional anonymous online Qualtrics survey was conducted at 4 dental schools in Michigan, Texas, Utah, Washington DC, and a single allopathic medical school in Michigan, in the United States between November 2021 and May 2022 as permission to access students at the different schools took varying amounts of time.

### Participants

Actively enrolled medical and dental students from each institution, 18 years of age or older, were eligible for the study. Eligible medical and dental students were contacted via email by a faculty member at each respective school on behalf of the authors and provided an information sheet and link to the survey. De-identified data was available to the authors only. A nominal incentive for participation was provided for survey completion by the study sponsor who did not have a role in the design, conduct, or data analysis of the study. This study was approved by the Oakland University Institutional Review Board and Howard University Review Board.

### Measures

In addition to demographic questions, the survey assessed general vaccine attitudes using the validated 5C Psychological Antecedents of Vaccination Scale with one item for each of the five C domains: Confidence, Complacency, Constraints, Calculation, and Collective Responsibility (10, 11). The 7-point Likert scale was recategorized into “agree” (strongly agree, moderately agree, agree), “disagree” (strongly disagree, moderately disagree, disagree), and neutral (mid-point on the Likert scale). Each item was individually recoded as accepting or hesitant (with neutral coded as hesitant).

Participants were asked about their personal vaccination status for the COVID-19 and influenza vaccines and answered vaccine-specific questions to assess knowledge, opinions, and behaviors, including comfort counseling patients and intent to administer these vaccines in their future practices. Vaccine-specific opinion and behavior items were developed based on past research and included Likert scale (strongly disagree to strongly agree) responses for the 12 opinion items and dichotomous (yes/no) responses for behavior items (12–14). Likert scale opinion items were then dichotomized as agree/disagree with neutral responses being coded as disagree. Opinion items were also summed to create an overall opinion score from 0 to 12 with a higher score representing more positive opinions. Investigator-generated vaccine-specific knowledge items were categorical (true/false/unsure) and scored as percent correct with unsure being coded as incorrect. A score of 80% or more was considered average/above-average knowledge. Time allotted to vaccine education in the curriculum was also asked (none/1–3 h/>3 h).

### Data analysis

Descriptive statistics (frequency and percentages) were performed for all variables and are presented for overall demographics and general vaccine attitudes. Chi-square was used to identify statistically significant differences between students with average/above-average knowledge and below-average knowledge and vaccine-specific opinions. Demographic and knowledge variables were included in logistic regression models to identify predictors for receiving each vaccine (COVID-19 or influenza), recommending the vaccine to patients, and providing the vaccine in the clinic. The same variables were included in multiple linear regression to identify predictors of overall COVID-19 and influenza vaccine behaviors and opinions.

## Results

Emails were sent out to 493 medical students and 1,316 dental students. A total of 232 medical students (response rate = 47%) and 221 dental students (response rate = 17%) responded to the survey, with 225 medical students and 202 dental students having complete data for analysis. The demographic distribution of the respondents was: 61% (*n* = 262) females, 48% (*n* = 206) aged 20–25 years, 45% (*n* = 192) aged 26–30 years, and 7% (*n* = 29) aged 31 years and older with 96% (*n* = 412) having had the COVID-19 vaccine and 86% (*n* = 367) having had the influenza vaccine.

### General vaccine attitudes (5C domains)

Overall, on the 5C domains, a majority of students (*n* = 402, 94%) reported that they were completely confident that vaccines are safe (confidence) and that they weigh the risks and benefits when deciding on vaccination (*n* = 329, 77%) (calculation). However, a noteworthy proportion of individuals reported barriers to vaccination, such as the perception that getting vaccinated is a hassle (*n* = 123, 29%) (constraints). A small proportion of students felt that vaccination is unnecessary because preventable diseases are not common (*n* = 21, 5%) (complacency) and that if everyone else is vaccinated they do not have to be vaccinated (*n* = 42, 10%) (collective responsibility).

### Vaccine knowledge

Two-hundred ninety-two students (68%) scored average/above-average knowledge on the COVID-19 vaccine knowledge items and 235 students (55%) scored average/above-average knowledge on the influenza vaccine knowledge items. Regarding the COVID-19 vaccine, 37% of the students incorrectly identified myocarditis as a common side effect, 33% thought that vaccine mRNA integrates with human DNA, and 30% thought that the vaccine was unsafe to use in pregnancy. Regarding the influenza vaccine, 19% of the students were unaware of the fact that the vaccine does not cause influenza and 36% did not know that the vaccine does not lead to long-lasting immunity. Half (50%) of the students were unaware of the contraindications of the nasal spray vaccine in pregnant patients.

### Curricular time

Nearly one-fifth (*n* = 77, 18%) of students reported having no training about vaccines in their curriculum with an additional 39% (*n* = 168) reporting only 1–3 h. Similarly, training in how to counsel on vaccines was minimal with nearly one-third (*n* = 133, 31%) reporting no training and 37% (*n* = 157) reporting 1–2 h.

### Vaccination status

COVID-19 vaccination status was significantly higher for students with average/above-average knowledge about the COVID-19 vaccine (70.4%) compared to those with below-average COVID-19 knowledge (29.6%). COVID-19-vaccinated students were 15.4 times more likely to have average/above-average knowledge than non-vaccinated students (95% CI 3.9–101.7). Similarly, influenza vaccination status was significantly higher for students with average/above- average knowledge about influenza vaccine (60.8%) compared to those with below-average influenza vaccine knowledge (39.2%). Influenza-vaccinated students were 6.2 times more likely to have average/above-average knowledge than non-vaccinated students (95% CI 3.2–12.5).

### Vaccine knowledge and opinions

Analysis of the association for vaccine knowledge for COVID-19 and influenza and respective opinions regarding counseling and future practice showed statistically significant differences for all opinion items (Table 1). Of note, for both vaccines, there were significant differences between average/above-average and below-average knowledge scores in terms of their roles as HCPs such as to learn about, recommend, and advocate for the respective vaccines as well as to counsel patients who are hesitant about the respective vaccine (*p* < 0.0001). Students with higher vaccine-specific knowledge also reported feeling having adequate knowledge to counsel about the vaccine (*p* < 0.0001). A number of students did not feel comfortable using evidence-based information to counter misinformation (18% for COVID-19 and 24% for influenza vaccine) or perceive themselves to have inadequate vaccine knowledge to counsel patients (22% for COVID-19 and 28% for influenza vaccine).

**Table 1:** Bivariate association between opinions and vaccine knowledge for COVID-19 and influenza (*n* = 427).

	COVID-19 vaccine specific knowledge			Influenza vaccine specific knowledge		
	Below average knowledge *n* (%) *n* = 135	Average-above average knowledge *n* (%) *n* = 292	Chi-square *p*-value	Below average knowledge *n* (%) *n* = 197	Average-above average knowledge *n* (%) *n* = 235	Chi-square *p*-value
It is my role as a healthcare provider to learn about “X” vaccines.	124 (91.8)	290 (99.3)	<0.0001	170 (89.4)	229 (97.5)	0.0008
It is my role as a healthcare provider to advocate for “X” vaccines.	82 (63.7)	285 (97.6)	<0.0001	150 (78.1)	224 (95.3)	<0.0001
I would recommend “X” vaccines to my friends and family.	90 (66.7)	288 (98.6)	<0.0001	158 (85.9)	223 (95.7)	0.0004
I would recommend “X” vaccines to my patients.	91 (67.4)	289 (99.0)	<0.0001	159 (82.8)	225 (95.7)	<0.0001
I would provide “X” vaccines in my clinic.	75 (55.6)	264 (90.4)	<0.0001	124 (64.6)	212 (90.2)	<0.0001
I believe the benefits of “X” vaccines outweigh the risks.	84 (62.2)	282 (96.7)	<0.0001	154 (85.4)	224 (95.3)	0.0004
It is important for healthcare providers to be trained to counsel their patients/parents about “X” vaccines.	101 (74.8)	286 (98.0)	<0.0001	167 (87.0)	225 (95.7)	0.001
It is important for healthcare providers to spend time counseling “X” vaccine-hesitant patients/parents.	85 (63.0)	284 (97.3)	<0.0001	141 (73.44)	220 (93.6)	<0.0001
“X” vaccines should be mandated for healthcare providers.	65 (48.2)	258 (88.4)	<0.0001	107 (55.7)	185 (78.7)	<0.0001
“X” vaccines should be mandated for the general public.	54 (40.0)	206 (70.6)	<0.0001	132 (68.8)	193 (82.1)	0.001
I feel I have the adequate knowledge required to counsel patients/parents with respect to “X” vaccines.	81 (60.0)	253 (86.6)	<0.0001	107 (55.7)	185 (78.7)	<0.0001
I feel comfortable using evidence-based information to counteract misinformation about “X” vaccines.	87 (64.4)	262 (89.7)	<0.0001	71 (37.0)	119 (50.6)	0.005

Table 2 reports the regression models and highlights the impact of COVID-19-specific and influenza-specific knowledge as a predictor of personal vaccine behavior, future practice regarding recommending or providing the vaccine, and overall opinions about the vaccine. In logistic regression controlling for age, gender, and profession, students with average/above-average knowledge of the COVID-19 vaccine were over seven times more likely to have received the vaccine compared to students with below-average knowledge (95% CI 1.44–35.87). When controlling for these variables and COVID-19 vaccination, students with average/above-average knowledge of the COVID-19 vaccine were 33 times more likely to recommend COVID-19 vaccine to patients (95% CI 8.02–140.94) and nearly 3 times more likely to provide the COVID-19 vaccine in their clinic (95% CI 1.51–5.12) compared to those with below-average knowledge of the COVID-19 vaccine.

**Table 2:** Logistic and multiple linear regression models predicting vaccine specific behavior and opinions (*n* = 427).

	Received vaccine^†^ OR (95% CI) (*p*)	Recommend vaccine to patients^a^ OR (95% CI) (*p*)	Provide vaccine in clinic^a^ OR (95% CI) (*p*)	Opinion score (0–12)^b^ Beta (SE) (*p*)
**COVID-19 vaccine**				
*Age*				
20–25 years	Ref.	Ref.	Ref.	Ref.
26–30 years	1.06 (0.29–3.90) (0.93)	0.99 (0.40–2.41) (0.98)	0.77 (0.42–1.42) (0.41)	0.41 (0.23) (0.08)
31+ years	0.26 (0.06–1.15) (0.08)	0.94 (0.21–4.16) (0.93)	3.76 (0.93–15.23) (0.06)	0.13 (0.49) (0.79)
*Gender*				
Male	0.81 (0.26, 2.54) (0.72)	0.18 (0.08–0.43) (0.0001)	0.61 (0.33–1.11) (0.11)	−0.75 (0.23) (0.001)
Female	Ref.	Ref.	Ref.	Ref.
*Profession*				
Medical students	5.21 (0.58–46.79) (0.14)	4.16 (1.21–14.28) (0.02)	13.98 (5.81–33.65) (<0.0001)	1.05 (0.26) (<0.0001)
Dental students	Ref.	Ref.	Ref.	Ref.
*Vaccine status*	–			
COVID-19 vaccine	–	46.74 (8.17–267.30) (<0.0001)	42.68 (4.55, 400.38) (0.001)	5.29 (0.64) (<0.0001)
No COVID-19 vaccine		Ref.	Ref.	Ref.
COVID-19 knowledge				
Avg-above average	7.18 (1.44–35.87) (0.02)	33.63 (8.02–140.94) (<0.0001)	2.78 (1.51–5.12) (0.001)	2.37 (0.27) (<0.0001)
Below average	Ref.	Ref.	Ref.	Ref.
**Influenza vaccine**				
*Age*				
20–25 years	Ref.	Ref.	Ref.	Ref.
26–30 years	1.84 (0.94–3.61) (0.07)	0.73 (0.33–1.62) (0.44)	0.58 (0.32–1.05) (0.07)	−0.51 (0.23) (0.02)
31+ years	0.42 (0.16–1.13) (0.09)	0.74 (0.23–2.38) (0.61)	2.59 (0.81–8.27) (0.11)	−0.01 (0.48) (0.99)
*Gender*				
Male	1.41 (0.72, 2.74) (0.32)	0.79 (0.37–1.70) (0.55)	1.49 (0.82–2.69) (0.19)	−0.20 (0.22) (0.38)
Female	Ref.	Ref.	Ref.	Ref.
*Profession*				
Medical students	31.10 (7.09–136.52) (<0.0001)	13.08 (2.76–61.90) (0.001)	14.10 (5.78–34.38) (<0.0001)	2.04 (0.27) (<0.0001)
Dental students	Ref.	Ref.	Ref.	Ref.
*Vaccine status*				
Influenza vaccine	–	5.84 (2.67–12.81) (<0.0001)	2.98 (1.51, 5.86) (0.002)	2.71 (0.35) (<0.0001)
No Influenza vaccine	–	Ref.	Ref.	Ref.
*Influenza knowledge*				
Avg-above average	2.02 (0.95–4.30) (0.07)	1.04 (0.44–2.49) (0.93)	1.32 (0.69–2.53) (0.39)	0.10 (0.26) (0.71)
Below average	Ref.	Ref.	Ref.	Ref.

^a^Logistic regression. ^b^Multiple linear regression.

In contrast, students with average/above-average knowledge of the influenza vaccine were not significantly more likely to have received the influenza vaccine (OR = 2.02, 95% CI 0.95–4.30), recommend influenza vaccine to patients (OR = 1.04, 95% CI 0.44–2.49), or provide influenza vaccine in their clinic (OR = 1.32 95% CI 0.69–2.53) compared to those with below-average knowledge of the influenza vaccine. Student profession was a significant predictor of recommending COVID-19 and influenza vaccines (COVID-19: OR = 4.16, 95% CI 1.21–14.28, influenza: OR = 13.08, 95% CI 2.76–61.90) and providing these vaccines in their clinic (COVID-19: OR = 13.98, 95% CI 5.81–33.65, influenza: OR = 14.10, 95% 5.78–34.38), with medical students significantly more likely to do these things compared to dental students. While student profession was a significant predictor of receiving the influenza vaccine (OR = 31.10, 95% CI 7.09–136.52) it was not for receiving the COVID-19 vaccine (OR = 5.21, 95% CI 0.58–46.79) (see Table 2).

In multiple linear regression controlling for age, gender, profession, and vaccination status, students with average/above-average knowledge about the COVID-19 vaccine had significantly more positive opinions about the COVID-19 vaccine compared to students with below-average knowledge (*p* < 0.0001). In contrast, there was no difference in opinions about the influenza vaccine by knowledge level (*p* = 0.71) (see Table 2).

## Discussion

Our study sheds light on the self-reported COVID-19 and influenza and vaccination behaviors, opinions, and knowledge of a large sample of future healthcare professionals: medical and dental students and confirms that students with better knowledge have more positive opinion should be revised to opinion about vaccines and future vaccine-specific behaviors in the clinical setting. However, in this group, detailed knowledge and perceptions of the vaccines are limited, as reported in previous studies (15).

We observed a positive relationship in this group of future HCPs between vaccine knowledge for COVID-19 and influenza and opinions regarding recommendations to patients, advocacy roles, and counseling vaccine hesitant patients. Our results suggest that these future HCPs with positive opinions towards vaccination are more likely to not only recommend vaccines to their patients but also spend time counseling vaccine hesitant patients. This is similar to findings in a systematic review by Lin et al. showing that HCPs who believed administering vaccination and advising patients about vaccines were their responsibility reported increased motivation to recommend the COVID-19 vaccine, discussed vaccines more often, and perceived greater vaccine utility (16).

Studies of vaccine hesitancy among HCPs show that HCPs are more likely to recommend vaccination to others if they are vaccinated (8). Lin et al. also report in their review that HCPs who received or planned to receive the influenza vaccine were 2.8–8 times more likely to recommend it (16). Our data revealed a higher vaccination rate among the medical students for both influenza and COVID-19 which was probably influenced by different vaccination requirements in hospital systems as compared to dental practices.

Research on the impact of vaccine-specific knowledge on vaccination behaviors and opinions concerning established versus newly developing vaccines in healthcare students is also limited. Our data indicate that students with high COVID-19 knowledge scores were significantly more inclined to recommend the COVID-19 vaccine to patients and willing to administer it within their clinics. However, this trend did not extend to their responses concerning the influenza vaccine. It is possible that these variations could be attributed to the long-established history of the influenza vaccine, which was devoid of the political and societal dynamics surrounding the COVID-19 vaccination during the survey period. Consequently, the impact of knowledge could pertain to vaccines that have been recently developed. Nonetheless, HCPS must stay current with knowledge and recommendations for routine and emergent vaccines. In a survey amongst healthcare providers in New York State, Fernandes et al. found that HCPs who correctly answered all four knowledge questions were more likely to self-report routine recommendations of standard vaccines to all patients when compared to those who correctly answered fewer questions (17). A meta-analysis by Paterson et al. also confirmed a positive relationship between providers’ knowledge about vaccines including knowledge about vaccine efficacy and safety and their willingness to recommend the vaccines (7).

A noteworthy finding in this study was that over one-third of these students have incorrect knowledge about COVID-19 and influenza vaccines, and that this gap in knowledge strongly impacted their opinions and ultimately their personal vaccine behaviors. This is unacceptable, as this lack of competence could result in incorrect patient education and management and these providers themselves are at a high risk of contracting these diseases from their patients. This knowledge deficit of key facts about these vaccines may be due to the minimal amount of time spent within the curriculum discussing vaccines as reported in the results of our study. We are also aware of the differences in curricular time spent teaching about immunity and vaccinations between medical and dental schools. Some areas need to be enhanced, as fair number of students who do not feel comfortable using evidence-based information to counter misinformation or perceive themselves to have inadequate vaccine knowledge to counsel patients. As previously noted, these results also show that students were more comfortable with COVID-19 vaccine counseling than with influenza. Educational goals should include increasing both students’ knowledge and competency in vaccine counseling. Knowledge gaps could be improved by devoting more time to vaccine teaching and enhancing communication skills with vaccine-hesitant patients. Vaccine hesitancy is a complex issue and teaching vaccine counseling requires a multifaceted approach that involves education, communication strategies, and ongoing adaptation. Providing appropriate frameworks for behavior change, such as the transtheoretical model, health belief model, or theory of planned behavior will allow future HCPs to counsel and guide vaccine-hesitant patients in a pro-active and nonjudgmental way and improve vaccine uptake (18, 19). It is imperative that future vaccine implementation strategies, particularly during public health crises, need to be targeted and a “one size fits all” is not adequate. We believe that knowledgeable HCPs will not only encourage vaccination in their practices but also be more engaged in their communities in the development and implementation of vaccine education initiatives.

### Limitations

Although this study was conducted at four dental and one medical school, the results may not extrapolate to all other medical and dental trainees in the country. Additionally, the number of unvaccinated students was very small for COVID-19 (*n* = 15, 4%) compared to influenza (*N* = 60, 14%) and could impact the findings. Furthermore, participation in the survey was voluntary, and this may have introduced a selection bias as students with greater interest in vaccination might have responded. While the incentive offered was minimal and not coercive, it could also have contributed to the selection bias of students who participated in the survey. This survey was conducted during the height of the COVID-19 pandemic and this may account for the differences in vaccine receipt, opinions, and future practices regarding the influenza vaccine as compared to the COVID-19 vaccine.

## Conclusion

HCPs play a crucial role in vaccine advocacy and public health. Equipping them with knowledge and communication skills will enable them to step into their role as strong vaccine advocates and improve health outcomes for themselves, their patients and their communities. This study highlights opinions about vaccination as well as gaps in knowledge in future dental and medical providers and provides relevant and actionable information regarding vaccine knowledge in medical and dental students. As medical and dental practitioners are at high risk for exposure to both influenza and COVID-19 in clinical practice, improved vaccine knowledge could be of personal benefit through increased vaccination uptake among this cohort.

Despite the limitations of the study, the findings provide a good foundation for curricular guidance and revision for medical and dental educators across the country in an effort to improve overall public health. Moving forward the lessons learned from this study could inform current approaches to teaching about boosters for the COVID-19 vaccine and also future pandemic responses and approaches to teaching HCP about new vaccines. Knowledge about emerging vaccines is crucial for advocacy and combating misinformation within the general population. Furthermore, being able to understand and clarify the scientific reasons for changing recommendations during the height of a public health emergency will empower patients to make more informed decisions about vaccine uptake during this time.

## Data availability statement

The raw data supporting the conclusions of this article will be made available by the authors, without undue reservation.

## Ethics statement

The studies involving humans were approved by Oakland University Institutional Review Board and Howard University Review Board. The studies were conducted in accordance with the local legislation and institutional requirements. The participants provided their written informed consent to participate in this study.

## Author contributions

VL: Conceptualization, Funding acquisition, Methodology, Writing – review & editing. AM: Formal analysis, Conceptualization, Funding acquisition, Methodology, Writing – review & editing. AK: Funding acquisition, Conceptualization, Methodology, Writing – review & editing. NA: Conceptualization, Funding acquisition, Methodology, Writing – original draft, Writing – review & editing.
